# Perceptions of research integrity climate differ between academic ranks and disciplinary fields: Results from a survey among academic researchers in Amsterdam

**DOI:** 10.1371/journal.pone.0210599

**Published:** 2019-01-18

**Authors:** Tamarinde L. Haven, Joeri K. Tijdink, Brian C. Martinson, Lex M. Bouter

**Affiliations:** 1 Department of Philosophy, Vrije Universiteit, Amsterdam, The Netherlands; 2 Department of Epidemiology and Biostatistics, Amsterdam University Medical Centers, Amsterdam, The Netherlands; 3 HealthPartners Institute, Research; Minneapolis Veterans Affairs Health Care System, Center for Care Delivery and Outcomes Research; University of Minnesota, Department of Medicine, Minneapolis, Minnesota, United States of America; London School of Economics and Political Science, UNITED KINGDOM

## Abstract

Breaches of research integrity have shocked the academic community. Initially explanations were sought at the level of individual researchers but over time increased recognition emerged of the important role that the research integrity climate may play in influencing researchers’ (mis)behavior. In this study we aim to assess whether researchers from different academic ranks and disciplinary fields experience the research integrity climate differently. We sent an online questionnaire to academic researchers in Amsterdam using the Survey of Organizational Research Climate. Bonferroni corrected mean differences showed that junior researchers (PhD students, postdocs and assistant professors) perceive the research integrity climate more negatively than senior researchers (associate and full professors). Junior researchers note that their supervisors are less committed to talk about key research integrity principles compared to senior researchers (*MD* = -.39, *CI* = -.55, -.24). PhD students perceive more competition and suspicion among colleagues (*MD* = -.19, *CI* = -.35, -.05) than associate and full professors. We found that researchers from the natural sciences overall express a more positive perception of the research integrity climate. Researchers from social sciences as well as from the humanities perceive less fairness of their departments’ expectations in terms of publishing and acquiring funding compared to natural sciences and biomedical sciences (*MD* = -.44, *CI* = -.74, -.15; *MD* = -.36, *CI* = -.61, -.11). Results suggest that department leaders in the humanities and social sciences should do more to set fairer expectations for their researchers and that senior scientists should ensure junior researchers are socialized into research integrity practices and foster a climate in their group where suspicion among colleagues has no place.

## Introduction

Recent breaches of research integrity in The Netherlands and worldwide have shocked the academic community [1–4]. Such events led to a new field of inquiry that aimed to better understand how common the problems are and what drives researchers to misbehave [5–7]. Initially, studies in this area mainly focused on research misconduct, in which there is generally an intent to deceive (fabrication, falsification, plagiarism). However, over time the focus broadened to the more frequent questionable research practices (QRPs). Accumulating empirical evidence has indicated QRPs are much more prevalent than formal research misconduct [8–10]. Consequently, QRPs probably have on the aggregated level more impact. Initially, explanations for research misconduct were sought at the level of individual researchers [11] but over time increased recognition emerged of the important role that structural and institutional factors such as research climate may play in influencing researchers’ behavior [12–16]. This has shifted the focus to the organizational climate in research settings as a potential target for intervention [17,18].

Studying organizational climates implies investigating the environment researchers work in and how this climate can strengthen or erode research integrity [19,20]. The organizational climate here is defined as “the shared meaning organizational members attach to the events, policies, practices, and procedures they experience and the behaviors they see being rewarded, supported, and expected.” (p. 115) [21,22]. Crain et al. [23] have documented that a favorable organizational research climate is positively associated with lower levels of self-reported questionable research practices. The Survey of Organizational Research Climate (henceforth: SOuRCe) is designed to measure the organizational research integrity climate in academic research settings [18,20,22,24].

The SOuRCe is embedded in two conceptual frameworks, the first being organizational justice theory [25]. In a nutshell: the fairer people regard decisions and decision-making processes in their organization, the more likely they trust their organization, abide by decisions made and do not engage in questionable behavior [26,27]. When people perceive procedural or distributional injustice in their organization, they are more likely to behave in ways that, in their mind, compensates for the perceived unfairness [27]. Applied to research integrity, in a research climate where perceived injustice is high, researchers would be expected to be more likely to engage in intentional research misconduct (falsification, fabrication and plagiarism) or questionable research practices [27].

The second conceptual framework underpinning the SOuRCe stems from the Institute of Medicine report *Integrity in Scientific Research*: *Creating an Environment That Promotes Responsible Conduct* [28]. This report describes the research environment as an open systems model where different factors influence research integrity. The report specifies that the research integrity climate can both stimulate or diminish responsible research [18,28,29]. Some key factors herein that are reflected in the SOuRCe are ethical leadership, integrity policy familiarization and communication, and the degree to which these are known by people in the organization [18,28].

Previous research with the SOuRCe found that researchers in different phases of their career perceive the research integrity climate differently [22]. PhD students perceived the climate to be fairer compared to senior scientists in that scholarly integrity was valued (e.g. acknowledging work of others). Senior scientists perceived there to be more resources for conducting research responsibly (e.g. policies to deal with integrity breaches were well known) [22].

Wells et al. [22] also found large differences in SOuRCe scores for different organizational subunits. Some had scores twice as negative compared to others or compared to overall mean scores. This indicates that overall high mean scores on an institutional level offer departmental leaders little comfort [20] and research climate may vary significantly within institutions. One factor that accounts for these stark differences between subunits was disciplinary field [22].

Our study aims to determine how scientists experience the research integrity climate, stratified for academic rank and disciplinary field, in two university medical centers and two universities in Amsterdam. This is the first study that investigates research integrity climate in The Netherlands. Assessing research integrity climate will provide insight what factors may hinder responsible research practices [26].

We hypothesized that we would observe significant variability in SOuRCe scale-scores based on (1) the disciplinary field in which academic researchers work and (2) the academic ranks of respondents. As our aim is descriptive in nature, we did not specify the direction of these differences.

## Materials and methods

### Ethical considerations

The Scientific and Ethical Review board of the Faculty of Behavior & Movement Sciences (Vrije Universiteit Amsterdam) approved our study (Approval Number: VCWE-2017-017R1).

### Participant selection and procedure

The institutions that participated in our study included two universities (Vrije Universiteit Amsterdam and University of Amsterdam) and two academic medical centers (Amsterdam Medical Centers). Upon securing endorsement from the deans and rectors of the participating institutions and finalizing a data sharing agreement, each institution provided a list of e-mail addresses of all researchers and PhD students. We distributed the electronic survey in May 2017 via email among all academic researchers. Researchers were eligible to participate if they were doing research at least one day per week (>0.2fte) on average. Our cross-sectional online survey contained three instruments (SOuRCe, the Publication Pressure Questionnaire [30] and a list of 60 major and minor research misbehaviors [9]). This article presents the SOuRCe results. The survey concluded with three demographic items about gender, academic rank and disciplinary field.

We used the online survey program Qualtrics (Qualtrics, Provo, UT, USA) to create and distribute the survey. Researchers first received an information e-mail explaining the purpose, goal and procedure of the study. After one week, we sent the official invitation with a unique link to the survey and a link to the non-response survey (see S1 Appendix). The invitation also included a link to our privacy policy and the protocol (see S2 Appendix and S1 Protocol), both available on the project’s website (www.amsterdamresearchclimate.com). The survey started with an online informed consent form. After consenting, participants were asked to indicate whether they were doing research for at least one day per week (inclusion criterion). We sent three reminders to those who had not responded yet. All correspondence explicitly stated that the data would remain confidential and that participation was voluntary.

### Instruments

We used the Survey of Organizational Research Climate [22–24,31]. The SOuRCe evaluates what factors play a role in the perceived research climate on a scale that ranges from 1 (“not at all”) to 5 (“completely”) [18,24]. It consists of 28 items forming 7 subscales that detail the organizational climate of integrity on a departmental and institutional level [29]. For an overview of the SOuRCe subscales, see Table 1.

**Table 1:** Overview of SOuRCe subscales.

Subscale	Level	# items	Description constructs measured
RCR Resources	Institutional	6	degree to which respondents perceive the existence of effective educational opportunities about RCR, available policies and professionals to whom concerns can be addressed, and leaders who actively support RCR
Regulatory Quality	Institutional	3	factors such as the degree to which regulatory committees such as the Medical Ethical Testing Committee treat researchers fairly.
Integrity Norms	Departmental	4	degree to which norms about research integrity exist in one’s department.
Integrity Socialization	Departmental	4	degree to which organizational departments engage in activities that effectively socialize junior researchers in research integrity.
Supervisor/Supervisee Relations	Departmental	3	relations between supervisors and their supervisees in terms of fairness, availability and respect
(Lack of) Integrity inhibitors*	Departmental	6	degree to which conditions like lack of adequate resources or suspicion and competition between colleagues produce difficulties for conducting research responsibly.
Expectations	Departmental	2	degree to which the department’s expectations for publishing and obtaining external funding are fair

Columns stipulate level of measurement, number of items per subscale and a description of the constructs that subscale measures.

*This scale was reversely scored so that all subscales can be interpreted using the same logic (i.e. a higher score means a greater lack of inhibiting factors, which indicates a better research integrity climate).

SOuRCe subscale scores are calculated by taking the average of all valid non-missing items in that subscale. The respondent needs to validly answer at least half of the items in the subscale for the subscale score to be valid. Valid scores are all response options except for “No basis for judging OR not relevant to my field of work”. All subscale scores can be interpreted using the same logic: the higher the score, the stronger the presence of that factor. Higher scores thus express a more favorable perception of the research integrity climate. While most SOuRCe items ask about the perceived presence of integrity supporting aspects of the local climate, the Integrity Inhibitors scale is comprised of items that ask about the perceived presence of factors that may inhibit research integrity. For analysis and reporting, the items contributing to this scale are reverse-coded so that the higher this subscale’s score; the greater the *lack* of integrity inhibiting conditions [29].

The SOuRCe was designed for a biomedical research setting. To make the items more applicable to all disciplinary fields from our study population, we slightly altered the wording of three items in consultation with the design team of the SOuRCe (see S1 Table). We also extended the response option: “No basis for judging” to “No basis for judging OR not relevant to my field of work”. Unfortunately, two of the original 28 SOuRCe items were inadvertently omitted from the final distribution of the questionnaire due to a programming error.

### Statistical analyses

The intended statistical analyses were preregistered under the title ‘Academic Research Climate Amsterdam’ at the Open Science Framework. Briefly, for the univariate analyses, we computed overall mean subscale scores and stratified scores per academic rank and disciplinary field. For those subscales where academic rank or disciplinary field was significantly associated, we tested whether stratified scores differed significantly using post hoc Bonferroni corrected *F* tests. We then created association models with academic rank or disciplinary field as independent variable and subscale score as dependent variable. For the multivariate analyses, we corrected for potential confounders (e.g. gender) or added effect modifiers when inspecting the relations between disciplinary field or academic rank and the SOuRCe subscales.

## Results

**Fig 1:**
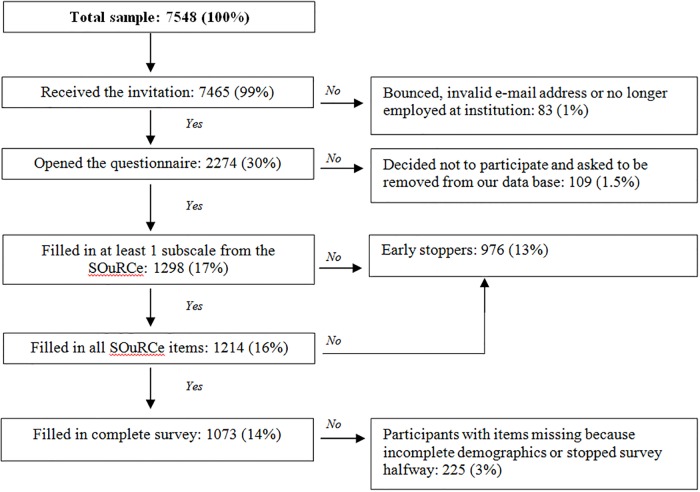
Flow diagram of response and completion rate. Percentages are expressed in reference to the total population of academic researchers in Amsterdam (*n* = 7548).

We collected 7548 e-mail addresses from academic researchers in Amsterdam. When we sent out the information letter, 83 bounced immediately as undeliverable. Also, 109 researchers decided not to participate and asked Qualtrics to be unsubscribed from the data base. 2274 researchers opened the questionnaire (30%). Of those who opened the questionnaire, 1298 (17% of the total sample) researchers answered enough questions to complete at least one SOuRCe subscale (57% of those who opened the questionnaire). See Fig 1. Only 2% filled in the ultra-brief non-response questionnaire.

### Differences between academic ranks

**Fig 2:**
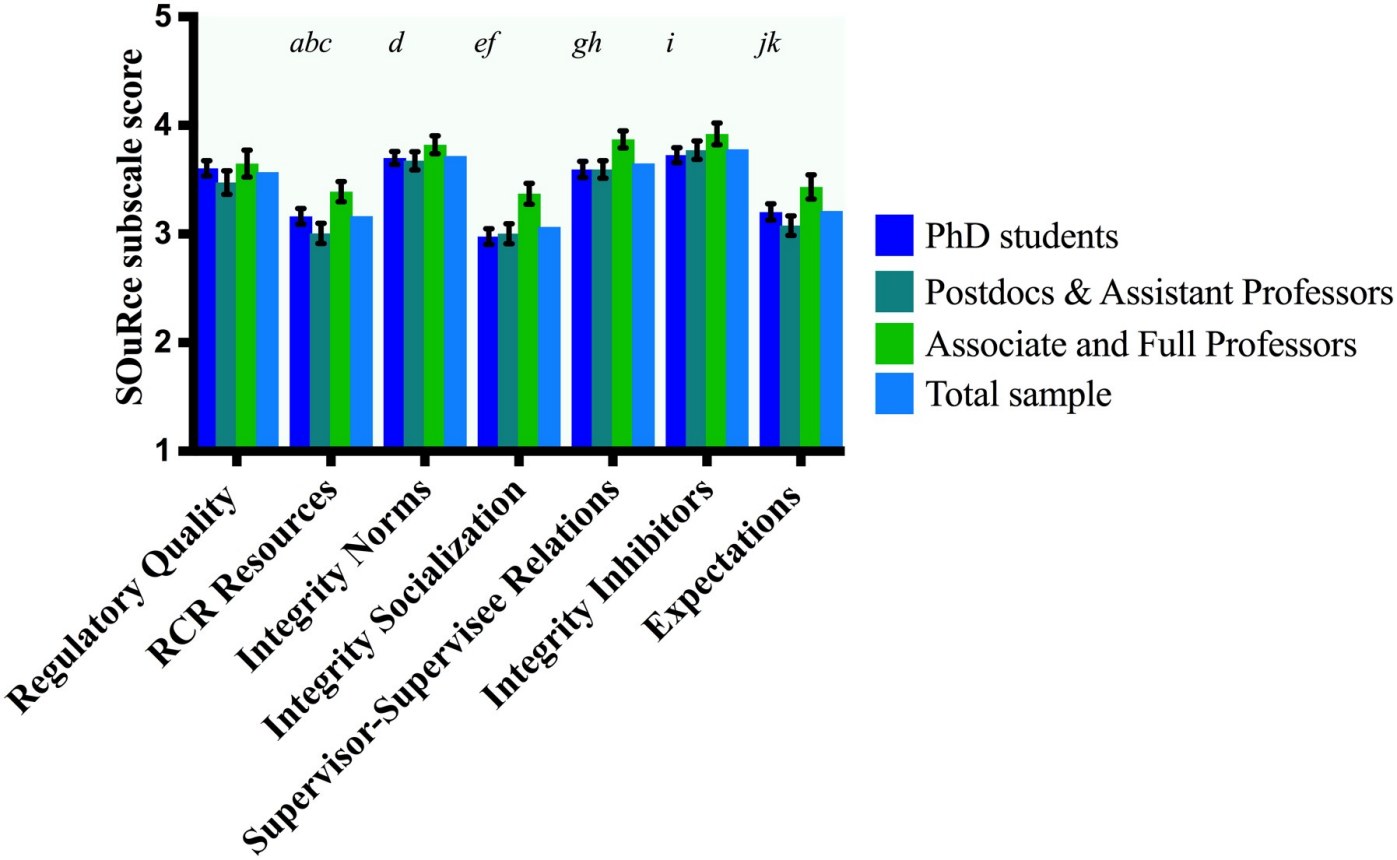
Differences between academic ranks. Gender adjusted (if applicable) and Bonferroni corrected mean differences (*MD*) between pairs of academic ranks on SOuRCe subscale scores with 95% confidence intervals (*CI*). Letters indicate significant differences at the α = 0.05 level. *a*: PhD students scored lower on RCR Resources than associate & full professors (*MD* = -.23, *CI* = -.39, -.07). *b*: Postdocs & assistant professors scored lower on RCR Resources than PhD students (*MD* = -.16, *CI* = -.3, -.02). *c*: Postdocs & assistant professors scored lower on RCR Resources than associate & full professors (*MD* = -.39, *CI* = -.56, -.21). *d*: Postdocs and assistant professors scored lower on Integrity Norms than associate & full professors (*MD* = -.15, *CI* = -.29, -.00). *e*: PhD students scored lower on Integrity Socialization than associate & full professors *(MD = -*.*39*, *CI* = -.55, -.24). *f*: Postdocs & assistant professors scored lower on Integrity Socialization than associate & full professors (*MD* = -.37, *CI* = -.54, .20). *g*: PhD students scored lower on Supervisor-Supervisee Relations than associate & full professors (*MD* = -.29, *CI* = -.43, -.14). *h*: Postdocs & assistant professors scored lower on Supervisor-Supervisee Relations than associate & full professors (*MD* = .28, *CI* = -.44, -.11). *i*: PhD students scored lower on Integrity Inhibitors than associate & full professors (*MD* = -.19, *CI* = -.35, -.05). *j*: PhD students scored lower on Expectations than associate & full professors (*MD* = -.23, *CI* = -.39, -.07). *k*: Postdocs & assistant professors scored lower on Expectations than associate & full professors (*MD* = -.36, *CI* = -.53, -.18).

**Fig 3:**
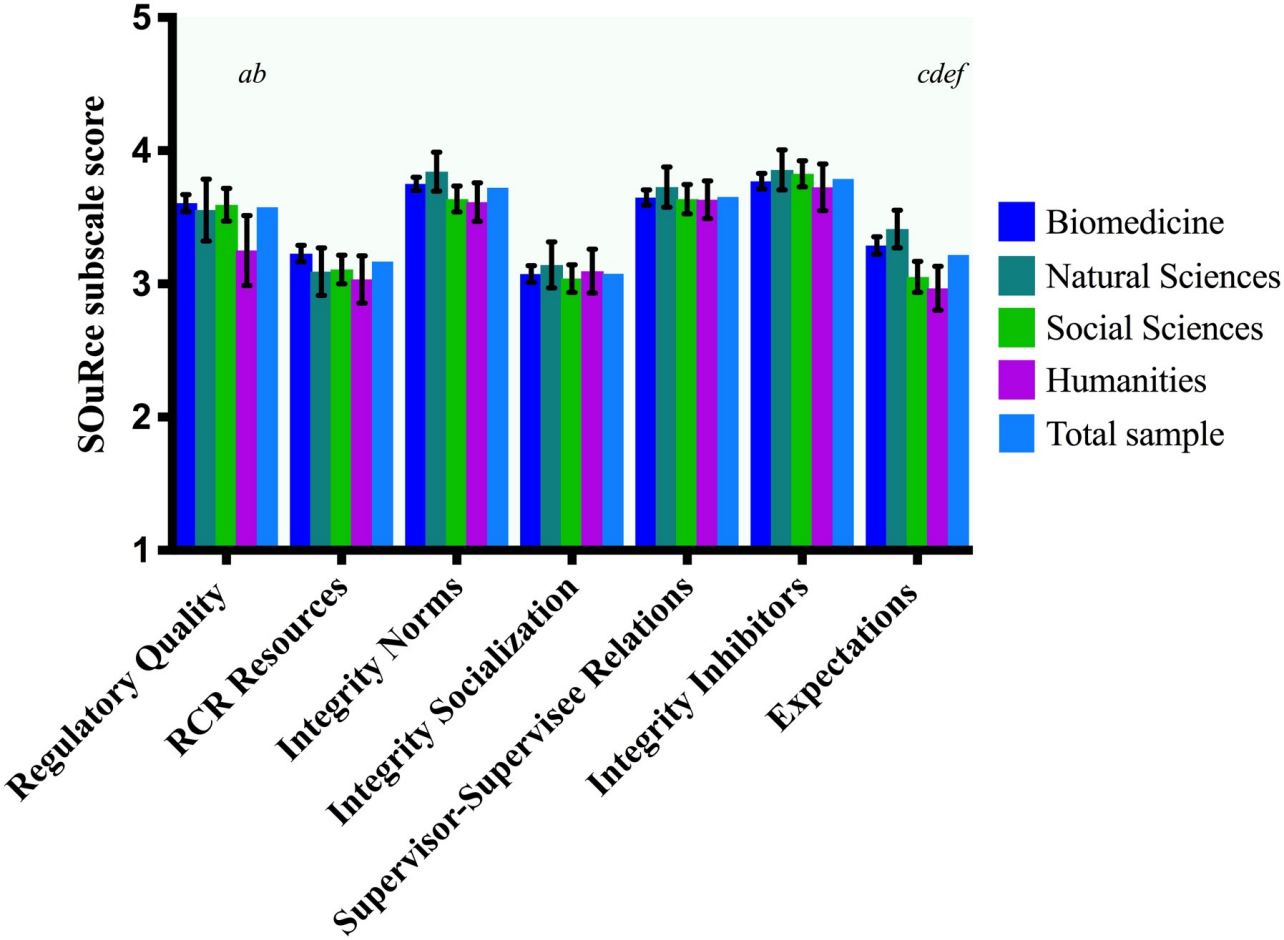
Differences between disciplinary fields. Rank adjusted (if applicable) and Bonferroni corrected mean differences (*MD*) between pairs of disciplinary fields on SOuRCe subscale scores with 95% confidence intervals (*CI*). Letters indicate significant differences at the α = 0.05 level. *a*: Humanities scored lower on Regulatory Quality than social sciences (*MD* = -.34, *CI* = -.66, -.01). *b*: Humanities scored lower on Regulatory Quality than biomedical sciences (*MD* = -.38, *CI* = -.68, -.08). *c*: Social sciences scored lower on Expectations than biomedical sciences (*MD* = .26, *CI* = -.44, -.09). *d*: Social sciences scored lower on Expectations than natural sciences (*MD* = -.38, *CI* = -.61, -.11). *e*: Humanities scored lower on Expectations than biomedical sciences (*MD* = -.34, *CI* = .57, -.10). *f*: Humanities scored lower on Expectations than natural sciences (*MD* = -.45, *CI* = -.75, -.15).

Overall mean subscale scores of the total sample are given in Figs 2 and 3 as a general reference to our stratified results. Investigating our first hypothesis (differences between academic ranks), for those subscales that were significantly associated with rank (Integrity Norms, Integrity Socialization, Integrity Inhibitors, Supervisor-Supervisee relations, Expectations and RCR Resources, respectively), we ran post-hoc Bonferroni corrected *F* tests. The purpose was to see whether PhD students, postdocs & assistant professors or associate & full professors perceived the climate differently on these subscale (see Table 2). PhDs students as well as postdocs and assistant professors scored significantly lower than associate and full professors on 4 subscales (Expectations, Supervisor-Supervisee relations, Integrity Socialization and RCR Resources, respectively). PhDs students (*M* = 3.73) also scored significantly lower than associate and full professors (*M* = 3.92) on Integrity Inhibitors. Postdocs and assistant professors (*M* = 3.67) scored significantly lower on Integrity Norms than did associate and full professors (*M* = 3.82) See Fig 2. Finally, we tested whether the relation between academic rank and the SOuRCe subscale scores was confounded or modified by other independent variables (i.e. gender or disciplinary field). Expectations and Integrity Norms were confounded by gender, respectively. Adding gender to these models made the associations between academic rank and SOuRCE subscale scores slightly weaker but the effect remained significant. We found effect modification by gender on RCR Resources only, these stratified results are given in Table 3. Therefore, Fig 2 and Table 2 display statistics corrected for confounding or reporting effect modification if applicable. We have calculated the effect sizes for the significant differences and interpreted these using Cohen [32], see Table 4.

**Table 2:** Regression models of SOuRCe subscales by academic rank.

Academic rank*	PhD students (*N* = 481)		Postdocs & assistant professors (*N* = 294)		Associate and full professors (*N* = 210) REF	
Scale *F* (*p*, *df*)	Beta (SE)	(CI)	Beta (SE)	(CI)	Beta (SE)	(CI)
**Integrity Norms** 3.21 (0.041, 2)	-.108 (.058)	(-.221, -.005)	-.138 (.062)	(-.259, -.016)	-	-
**RCR Resources** 14.043 (< .001, 2)	-.386 (.102)	(-.586, -.186)	-.476 (.110)	(-.691, -.260)	-	-
**Integrity Inhibitors** 4.908 (.008, 2)	-.195 (.063)	(-.317, -.073)	-.150 (.068)	(-.283, -.017)	-	-
**Integrity Socialization** 19.584 (< .001, 2)	-.394 (.065)	(-.522, -.266)	-.368 (.071)	(-.507, -.228)	-	-
**Supervisor-Supervisee Relations** 11.552 (< .001, 2)	-.284 (.062)	(-.405, -.162)	-.278 (.068)	(-.411, -.144)	-	-
**Expectations** 11.772 (< .001, 2)	-.202 (.070)	(-.340, -.064)	-.335 (.075)	(-.482, -.189)	-	-

Regression coefficients (Beta), standard errors (SE) and confidence intervals (CI). F-tests (*F*) between groups are given in the left column associated *p*-value and degrees of freedom (*df*).

* 313 respondents did not disclose their academic rank.

**Table 3:** Stratified scores on RCR Resources for academic rank and gender.

Academic rank	Male	Female
PhD student	3.29	3.10
Postdoc and Assistant Professor	2.99	3.01
Associate and Full Professor	3.33	3.50

**Table 4:** Overview of effect sizes of significant differences (p < .05).

Subscale SOuRCe	Group vs. Group	Effect size*	Interpretation**
**RCR Resources**	PhD students < Associate & full professors	.29	Small
**RCR Resources**	Postdocs & assistant professors < PhD students	.19	Small
**RCR Resources**	Postdocs & assistant professors < Associate & full professors	.47	Small
**Integrity Norms**	Postdocs & assistant professors < Associate & full professors	.22	Small
**Integrity Socialization**	PhD students < Associate & full professors	.18	Small
**Integrity Socialization**	Postdocs & assistant professors < Associate & full professors	.87	Large
**Supervisor-Supervisee Relations**	PhD students < Associate & full professors	.36	Small
**Supervisor-Supervisee Relations**	Postdocs & assistant professors < Associate & full professors	.43	Small
**Integrity Inhibitors**	PhD students < Associate & full professors	.25	Small
**Expectations**	PhD students < Associate & full professors	.27	Small
**Expectations**	Postdocs & assistant professors < Associate & full professors	.43	Small
**Regulatory Quality**	Humanities < Biomedical sciences	.50	Medium
**Regulatory Quality**	Humanities < Social sciences	.42	Small
**Expectations**	Social sciences < Biomedical sciences	.29	Small
**Expectations**	Social sciences < Natural sciences	.42	Small
**Expectations**	Humanities < Biomedical sciences	.39	Small
**Expectations**	Humanities < Natural sciences	.55	Medium

* based on Hedges’ *G* that is calculated as: M1-M2SDpooled)

** An effect size of .20 is small, .50 is medium, .80 is large and 1.30 is very large.

### Differences between disciplinary fields

Regarding our second hypothesis (differences between disciplinary fields), disciplinary field was associated with Regulatory Quality and Expectations, see Table 5. Humanities scored significantly lower on Regulatory Quality than biomedicine. Social Sciences (*M* = 3.05) as well as humanities (*M* = 2.97) score significantly lower on Departmental Expectations than both natural sciences (*M* = 3.41) and biomedicine (*M* = 3.29). See Table 5 and Fig 3. The associations between disciplinary field and both Regulatory Quality as well as Expectations were confounded by rank, yet again the main effect of discipline remained significant. Therefore, Fig 3 and Table 5 display statistics corrected for confounding. We have calculated the effect sizes of each difference, see Table 4.

**Table 5:** Regression models of SOuRCe subscales by disciplinary field.

Disciplinary field*	Biomedical sciences (*N* = 557)		Natural sciences (*N* = 103)		Social sciences (*N* = 237)		Humanities (*N* = 100) REF	
Scale *F* (*p*, *df*)	Beta (SE)	(CI)	Beta (SE)	(CI)	Beta (SE)	(CI)	Beta (SE)	(CI)
**Regulatory quality** 3.472 (.016, 3)	.395 (.113)	(.174, .617)	.391 (.164)	(.070, .711)	.395 (.125)	(.150, .640)	-	-
**Expectations** 9.709 (< .001, 3)	.366 (.089)	(.193, .540)	.483 (.113)	(.261, .705)	.142 (.099)	(-.052, .337)		

Regression coefficients (Beta), standard errors (SE) and confidence intervals (CI). F-tests (*F*) between groups are given in the left column associated *p*-value and degrees of freedom (*df*).

* 281 respondents did not disclose their disciplinary field.

## Discussion

We assessed the research integrity climate in Amsterdam using the SOuRCe. We hypothesized that we would observe significant variability in SOuRCe scale-scores based on (1) the disciplinary field in which academic researchers work and (2) the academic ranks of respondents. For the sake of brevity, we therefore discuss only the significant differences between academic ranks and disciplinary fields below.

### Differences between academic ranks

Departmental Expectations were perceived more negatively by PhD students, postdocs and assistant professors. This could be because their career prospects often directly depend on fulfilling these expectations whereas more senior scientists are less directly dependent on meeting publication and funding requirements for retaining their job [33,34]. This result is similar to Martinson et al. (2006) who found mid- and early career scientists to perceive higher amounts of organizational injustice compared to senior scientists as measured by asking scientists about the efforts they put into scientific work and rewards they receive in return [35–37].

We found PhDs as well as postdocs and assistant professors to score lower on Supervisor-Supervisee relations than associate and full professors. Martinson et al. [20] found the opposite effect in their study of researchers within the U.S. Department of Veterans Affairs Healthcare System in which the senior staff perceived this scale to be lower than more junior staff. In contrast, in a study of more traditional academic researchers in the U.S., Wells et al. [22] did not find notable differences on this scale by academic rank. The fact that junior researchers in our sample perceive their supervision as suboptimal could be alarming as poor mentoring is associated with the risk of emotional stress [38,39] and poor mentoring is viewed by some as one of the most impactful research misbehaviors [9].

Contrary to Wells et al. [22] who found U.S. junior researchers to report the highest levels of Integrity socialization, we found PhDs and postdocs to report lower levels of Integrity socialization than professors. Junior researchers are the ones who would have to be ‘socialized’ into research integrity whereas senior researchers in charge of this socialization process report higher levels. This discrepancy could indicate that senior researchers acknowledge the importance of research integrity when it comes to effective socialization of junior researchers into the department, yet we may conclude that in practice this socialization into research integrity does not get sufficient attention.

Communication about research integrity policies, part of the RCR Resources subscale, from the various bodies in academia is often addressed to the deans, department heads or principal investigators. This could explain why Wells et al. [22] found the same result as we have here: senior researchers score higher on RCR Resources than junior researchers. Being a senior researcher (associate & full professor) in an academic organization inevitably means that research integrity policies created at the top are more likely to land on your desk.

Interestingly, this effect depended on gender: female researchers perceived more RCR Resources, except for PhD students where male researchers perceived more resources to conduct their research responsibly. Perhaps female PhD students are also more likely to express their concern about the availability of resources for responsible conduct of research than their male counterparts. There is some evidence that women value procedural justice, the way in which resources are distributed, more than men do [40] but as no gender interactions in SOuRCe subscales have been reported, it seems premature to conclude that this applies here.

PhD students perceived the [lack of] Integrity Inhibitors to be lower than did associate and full professors. Mirroring the pattern for this subscale of Wells et al. [22], PhD students perceive a larger presence of such integrity inhibiting conditions (such as suspicion among colleagues or a hostile atmosphere) than more senior researchers. Associate and full professors may have gotten used to inhibitors such as publication pressure and regard these as less of a threat to integrity [22,41].

Finally, postdocs and assistant professors perceive Integrity Norms to be lower than associate and full professors, indicating a more negative attitude towards research integrity. Maybe postdocs and assistant professors witness less responsible research and more QRPs. This again parallels the three U.S. universities findings where postdocs scored lowest on more than half of the SOuRCe subscales [22]. Postdocs and assistant professors perceiving more questionable conduct of research also aligns with studies assessing the frequency of misbehavior, where mid-career scientists admitted to more research misbehaviors than did senior scientists [5].

### Differences between disciplinary fields

Similar to Wells et al. [22], we found the humanities to score lowest on both Departmental Expectations and Regulatory Quality. The difference was to be expected as regulatory bodies play a more important role in fields where rules and regulation are pivotal (such as biomedicine). In areas like literature or philosophy, regulatory bodies are less important or non-existent. Hence, researchers from the humanities might score lower because they do not encounter these regulating bodies.

The subscale Departmental Expectation measures the degree to which researchers perceive their department’s expectations regarding publishing or obtaining funding as fair. Alike Wells et al. [22] natural sciences score highest and the humanities score lowest. One explanation could be that in areas like philosophy or law the traditional way of disseminating academic work is via books, national or specialist journals. Nowadays when performance is measured the focus is predominantly on publishing in (high-impact) international journals. This can cause dissatisfaction from researchers from the humanities, as their books and national contributions are not valued the same way by their department as other academic products such as journal publications.

### Strengths of our study

Ours is the first publicly available study to investigate the research integrity climate in a European country. It is too premature to compare our data to the U.S. studies available, as differences in research integrity climates found could be due to a range of factors (known and unknown) that neither of these studies has measured. Our data can provide a useful baseline measurement so that repeated administration of the SOuRCe could provide information on developments over time. With this knowledge we can better inform universities about interventions tailored to specific disciplines and ranks. This can be used to create a better climate for research integrity.

Furthermore, the SOuRCe subscales focus on observable characteristics in the local environment. This means that the SOuRCe provides direct feedback for academic leaders on what can be improved in the organizational structure for fostering research integrity. For example, we found Integrity Socialization is perceived low by junior researchers. This result might target investigation at the institution to find out how socialization can be boosted, how and what means are necessary to foster embedding of research integrity socialization.

### Study limitations

Although our completion rate of 18% is low, it is similar to other online surveys. This does not necessarily indicate response bias [42,43]. Response bias could occur when non-responders are dissimilar to responders. We tried to estimate this by asking non-responders to fill in a brief non-response questionnaire, but only 2% of non-responders did which we regard too little to base solid conclusions on. We thus tried to assess the representativeness of our respondents for the total population by comparing our demographics to publicly available data on researchers in Amsterdam. Because data on researchers in medical centers is not readily available, we decided to filter out all researchers who indicated working in biomedical sciences. When comparing the researchers in our sample from the two universities (excluding all researchers from the two medical centers) to the publicly available data on researchers at the two universities in Amsterdam, it appears that we had a reasonably representative sample taking part from the various ranks: 27% of researchers are full or associate professor (our sample: 21%), 40% are assistant professor or postdoc (our sample 38%) and 32% are PhD-student (our sample: 41%).

However, there may be a gender bias as more than half of the researchers in our sample were female (57% respectively). In the Netherlands as a whole, females only account for 39% of academics (https://www.vsnu.nl/f_c_ontwikkeling_aandeel_vrouwen.html). In Amsterdam, that is 42%. This is most likely accounted for by an overrepresentation of female PhD students in our sample (68% versus the national 45% of PhD students in academia). This could be due to women’s greater willingness to participate in surveys [44,45]. However, we accounted for this selectivity by correcting for gender where necessary. In the case of RCR Resources, gender modified the results. Hence, we report this effect separately for men and women (see Table 3). To conclude, this selectively of the sample is unlikely to bias our results.

Also, to protect respondents’ and institutions’ privacy, we decided to only collect personal information about gender, academic rank and disciplinary field. This restricted our ability to obtain institutional-level, department-level and specific field of study level classifications, making it likely that we have missed meaningful variability between institutes or departments within our broad disciplinary categories. This way of collecting our data on relatively large group level only (academic rank and disciplinary field) also makes a more advanced multilevel model infeasible, so results from our multivariate association models (see Tables 2 and 5) should be interpreted with caution as the standard errors of observed associations may be under-estimated due to clustering [46]. We tried to estimate the impact of clustering using unpublished ICCs from the data used by Wells et al. [22] for institute (they had three participating institutions, we have four). Applying the clustering correction affected the relation with rank and Integrity Norms and Integrity Inhibitors: rank was no longer significantly associated with these three subscales. Other associations with rank remained significant despite the VIF correction, see S2 Table. Disciplinary field remained significantly associated with both Expectations and Regulatory Quality, see S3 Table).

### Implications

The core finding that the research integrity climate is perceived differently by juniors and seniors as well as by researchers from different disciplinary fields, stresses the need for tailored interventions. A one-size-fits all approach to improve the academic research integrity climate will likely not yield the desired effect [23]. Interestingly, nowadays more attention is paid to proper research integrity education via means of tutorials, seminars and other courses. This does not align with the low score on Integrity Socialization and RCR resources in our sample. However, integrity is not something someone learns from one course, responsible research has to become a habit, not an exception. There is terrain to win by integrating research integrity into daily practice by taking time to make every new researcher in the department familiar with research integrity. Furthermore, it can help to focus discussions about research integrity on the actual situation in the department: what standard procedures have been implemented to foster responsible research without having to compromise research integrity.

A rather alarming observation in our results is PhD students’ perception of integrity inhibiting factors. The novices in academic research already have to cope with suspicion and competition among colleagues. Navigating in a research integrity climate with such challenges asks for thoughtful guidance from senior researchers that sadly seems no to have no priority [9].

In conclusion, the research integrity climate is perceived differently by researchers from different disciplinary fields. Small fields like the humanities perceive their department’s expectations as more negative compared to other disciplinary fields. The natural sciences overall seem to perceive the climate more positively.

Associate and full professors perceive a more positive research integrity climate than assistant professors, postdocs and PhD-students. This might be a key for improving the research integrity climate. Senior scientists should ensure that new researchers are socialized into research integrity practices and foster a climate in their group where suspicion among colleagues has no place.

## Supporting information

S1 Appendix Non-response survey. (PDF)

S2 Appendix Privacy policy academic research climate Amsterdam. (PDF)

S1 Protocol Academic research climate Amsterdam protocol. (PDF)

S1 Table Modifications in SOuRCe items for current study. (PDF)

S2 TableVariance Inflation correction tests for association models with academic rank.Clustering refers to situations where there is non-independence of observations in the data, resulting in “design effects” or “intraclass-correlations” (ICCs) in the data. In our study, respondents are clustered (or nested) in departments, that are again nested within disciplines that are themselves nested within institutions introducing dependence in the data on different levels. Inference on regression coefficients needs to take this dependence into account. Ignoring the clustering in the analyses yields estimates for the standard errors for the betas that are too small, and hence, will also result in p-values that are too small (and increase of type I-errors). For reasons of privacy, data concerning affiliation of the respondents was not available and for this reason a standard multilevel analysis correcting for clustering could not be performed. We therefore used a linear regression with a post-hoc correction of the SE’s of the beta’s using an estimate for the Variance Inflation Factor. By correcting the SE’s, we get some indication of whether the associations we found are still there had we taken clustering into account [46]. The authors of the Wells et al. [22] study calculated their *ICC institute* (see *ICC institute* in the formulae below) for us. This way we could compute the Variance Inflation Factor as a correction for the sample size we used to estimate our SE’s. We re-calculated the t-statistics, using adjusted degrees of freedom and adjusted SE’s, to assess whether the associations we found between rank or discipline and the SOuRCe subscales are also detected when clustering is taken into account. The calculations are according to the following formulas:
VIF=1+(m-1)*ICCinstitute
$$CorrectedSE=SD/\sqrt{(n}/VIF)$$
$$t=\frac{\beta }{CorrectedSE}$$
$Df\;=\;\frac{N}{VIF}-p-1$The left column describes the relevant SOuRCe subscale with the effective *N*, the second column the VIF value based on the ICC for Institute from the Wells et al. study, then the adjusted t-scores and significance level of the corrected association is given. The final right column concludes what the impact of the VIF correction meant for the initially found association.(PDF)

S3 Table Variance Inflation correction tests for association models with disciplinary field. Clustering refers to situations where there is non-independence of observations in the data, resulting in “design effects” or “intraclass-correlations” (ICCs) in the data. In our study, respondents are clustered (or nested) in departments, that are again nested within disciplines that are themselves nested within institutions introducing dependence in the data on different levels. Inference on regression coefficients needs to take this dependence into account. Ignoring the clustering in the analyses yields estimates for the standard errors for the betas that are too small, and hence, will also result in p-values that are too small (and increase of type I-errors). For reasons of privacy, data concerning affiliation of the respondents was not available and for this reason a standard multilevel analysis correcting for clustering could not be performed. We therefore used a linear regression with a post-hoc correction of the SE’s of the beta’s using an estimate for the Variance Inflation Factor. By correcting the SE’s, we get some indication of whether the associations we found are still there had we taken clustering into account [46]. The authors of the Wells et al. [22] study calculated their *ICC institute* (see *ICC institute* in the formulae below) for us. This way we could compute the Variance Inflation Factor as a correction for the sample size we used to estimate our SE’s. We re-calculated the t-statistics, using adjusted degrees of freedom and adjusted SE’s, to assess whether the associations we found between rank or discipline and the SOuRCe subscales are also detected when clustering is taken into account. The calculations are according to the following formulas: The left column describes the relevant SOuRCe subscale with the effective N, the second column the VIF value based on the ICC for Institute from the Wells et al. [22] study, then the adjusted t-scores and significance level of the corrected association is given. The final right column concludes what the impact of the VIF correction meant for the initially found association.
(PDF)

## References

[1] Bouter LM. Commentary: Perverse incentives or rotten apples? Account Res. 2015;22(3):148–61. 10.1080/08989621.2014.950253 25635847

[2] Levelt Committee, Noort Committee, Drenth Committee. Flawed science: The fraudulent research practices of social psychologist Diederik Stapel. 2012.

[3] Stroebe W, Postmes T, Spears R. Scientific misconduct and the myth of self-correction in science. Perspect Psychol Sci. 2012;7(6):670–88. 10.1177/1745691612460687 26168129

[4] Kornfeld DS. Perspective: Research misconduct: The search for a remedy. Acad Med. 2012;87(7):877–82. 10.1097/ACM.0b013e318257ee6a 22622208

[5] Martinson BC, Anderson MS, de Vries R. Scientists behaving badly. Nature. 2005;435(7043):737–8. 10.1038/435737a 15944677

[6] Titus SL, Wells JA, Rhoades LJ. Repairing research integrity. Nature. 2008;453(7198):980–2. 10.1038/453980a 18563131

[7] Fanelli D. How many scientists fabricate and falsify research? A systematic review and meta-analysis of survey data. PLoS One. 2009;4(5):e5738 10.1371/journal.pone.0005738 19478950 PMC2685008

[8] Steneck N. Fostering integrity in research: Definition, current knowlege, and future directions. Sci Eng Ethics. 2006;12(1):53–74. 16501647 10.1007/pl00022268

[9] Bouter LM, Tijdink J, Axelsen N, Martinson BC, ter Riet G. Ranking major and minor research misbehaviors: results from a survey among participants of four World Conferences on Research Integrity. Res Integr Peer Rev. 2016;1(17):1–8. 29451551 10.1186/s41073-016-0024-5 PMC5803629

[10] De Vries R, Anderson MS, Martinson BC. Normal misbehavior: scientists talk about the ethics of research. J Empir Res Hum Res Ethics. 2006;1(1):43–50. 10.1525/jer.2006.1.1.43 16810336 PMC1483899

[11] Shaw D. The quest for clarity in research integrity: A conceptual schema. Sci Eng Ethics. 2018; 10.1007/s11948-018-0052-2 29594670

[12] Casadevall A, Ellis LM, Colloquium AAM, Committee S, Davies EW, Manager PE, et al A framework for improving the quality of research in the biological sciences. MBio. 2016;7(4):e01256–16. 10.1128/mBio.01256-16 27578756 PMC4999552

[13] Sovacool BK. Exploring scientific misconduct: Isolated individuals, impure institutions, or an inevitable idiom of modern science? J Bioeth Inq. 2008;5(4):271–82.

[14] Steneck NH. Institutional and individual responsibilities for integrity in research. Am J Bioeth. 2002;2(4):51–3. 10.1162/152651602320957574 12762926

[15] Casadevall A, Fang FC. Reforming science: Methodological and cultural reforms. Infect Immun. 2012;80(3):891–6. 10.1128/IAI.06183-11 22184414 PMC3294645

[16] Alberts B, Kirschner MW, Tilghman S, Varmus H. Rescuing US biomedical research from its systemic flaws. Proc Natl Acad Sci. 2014;111(16):5773–7. 10.1073/pnas.1404402111 24733905 PMC4000813

[17] Martinson BC, Mohr DC, Charns MP, Nelson D, Hagel E, Bangerter A, et al Main Outcomes of an RCT to Pilot Test Reporting and Feedback to Foster Research Integrity Climates in the VA. AJOB Empir Bioeth. 2017;8(3):211–9. 10.1080/23294515.2017.1363318 28949895 PMC5689383

[18] Thrush CR, Vander Putten J, Gene Rapp C, Pearson C, Simms Berry K, O’Sullivan P. Content validation of the Organizational Climate for Research Integrity survey (OCRI). J Empir Res Hum Res Ethics. 2007;2(4):35–52. 10.1525/jer.2007.2.4.35 19385806

[19] Mumford M, Murphy S, Connelly S, Hill J, Antes A, Brown R, et al Environmental influences on ethical decision making: climate and environmental predictors of research integrity. Ethics Behav. 2007;17(4):337–66.

[20] Martinson BC, Nelson D, Hagel-Campbell E, Mohr D, Charns MP, Bangerter A, et al Initial results from the Survey of Organizational Research Climates (SOuRCe) in the U.S. department of veterans affairs healthcare system. PLoS One. 2016;11(3). 10.1371/journal.pone.0151571 PMC4788347 26967736

[21] Schneider B, Ehrhart MG, Macey WH. Organizational Climate and Culture. Annu Rev Psychol. 2013;64(1):361–88. 22856467 10.1146/annurev-psych-113011-143809

[22] Wells JA, Thrush CR, Martinson BC, May TA, Stickler M, Callahan EC, et al Survey of organizational research climates in three research intensive, doctoral granting universities. J Empir Res Hum Res Ethics. 2014;9(5):72–88. 10.1177/1556264614552798 25747692

[23] Crain AL, Martinson BC, Thrush CR. Relationships between the Survey of Organizational Research Climate (SORC) and self-reported research practices. Sci Eng Ethics. 2013;19(3):835–50. 10.1007/s11948-012-9409-0 23096774 PMC3594440

[24] Martinson BC, Thrush CR, Crain AL. Development and validation of the Survey of Organizational Research Climate (SORC). Sci Eng Ethics. 2013;19(3):813–134. 10.1007/s11948-012-9410-7 23096775 PMC3594655

[25] Greenberg J. Allocator-recipient similarity and the equitable division of rewards. Soc Psychol (Gott). 1978;41(4):337–41.

[26] Martinson BC, Anderson MS, Crain AL, De Vries R. Scientists’ perceptions of organizational justice and self-Reported misbehaviors. J Empir Res Hum Res Ethics. 2006;1(1):51–66. 10.1525/jer.2006.1.1.51 16810337 PMC1483900

[27] Martinson BC. The Importance of Organizational Justice in Ensuring Research Integrity. J Empir Res Hum Res Ethics. 2010;5(3):67–83. 10.1525/jer.2010.5.3.67 20831422 PMC3032394

[28] IOM. Integrity in scientific research: Creating an environment that promotes responsible conduct. Washington, D.C.: National Academy of Sciences; 2002. 24967480

[29] Thrush CR, Martinson BC, Crain AL, Wells JA. User ‘ s Manual Survey of Organizational Research Climate. 2011;

[30] Tijdink J, Smulders Y, Vergouwen A, de Vet H, Knol DL. The assessment of publication pressure in medical science; validity and reliability of a Publication Pressure Questionnaire (PPQ). Qual life Res An Int J Qual life Asp Treat care Rehabil. 2014;23(7):2055–62. 10.1007/s11136-014-0643-6 24522963

[31] Martinson BC, Nelson D, Hagel-Campbell E, Mohr D, Charns MP, Bangerter A, et al Initial results from the Survey of Organizational Research Climates (SOuRCe) in the U.S. department of veterans affairs healthcare system. PLoS One. 2016;11(3):1–18. 10.1371/journal.pone.0151571 PMC4788347 26967736

[32] Cohen J. Statistical power analysis for the behavioral sciences. 2nd ed Hillsdale, N.J.: Erlbaum; 1995.

[33] Van Dalen Hendrik; Henkens K. Intended and unintended consequences of a publish-or-perish culture: A worldwide survey. J Am Soc Inf Sci Technol. 2012;63(7):1282–1293.

[34] Mentzelopoulos SD, Zakynthinos SG. Research integrity, academic promotion, and attribution of authorship and nonauthor contributions. JAMA—J Am Med Assoc. 2017;318(13):1221–2. 10.1001/jama.2017.11790 28880990

[35] Peter R, Alfredsson L, Hammar N, Siegrist J, Theorell T, Westerholm P, et al High effort, low reward, and cardiovascular risk factors in employed Swedish men and women : baseline results from the WOLF Study. J Epidemiol Community Heal. 1998;52:540–7. 10.1136/jech.52.9.540 PMC1756758 10320854

[36] Siegrist J. Adverse health effects of high-effort/low-reward conditions. J Occup Health Psychol. 1996;1(1):27–41. 9547031 10.1037//1076-8998.1.1.27

[37] Siegrist. Effort-reward imbalance at work and health. Hist Curr Perspect Stress Heal. 2015;2:261–91.

[38] Peluso DL, Carleton RN, Asmundson GJG. Depression symptoms in Canadian psychology graduate students: Do research productivity, funding, and the academic advisory relationship play a role? Can J Behav Sci. 2011;43(2):119–27.

[39] Levecque K, Anseel F, De Beuckelaer A, Van der Heyden J, Gisle L. Work organization and mental health problems in PhD students. Res Policy. 2017;46(4):868–79.

[40] Sweeney PD, Mcfarlin DB. Process and outcome: Gender differences in the assessment of justice. J Organ Behav. 1997;18(1):83–98.

[41] Tijdink JK, Schipper K, Bouter LM, Pont PM, De Jonge J, Smulders YM. How do scientists perceive the current publication culture? A qualitative focus group interview study among Dutch biomedical researchers. BMJ Open. 2016;6(2). 10.1136/bmjopen-2015-008681 PMC4762115 26888726

[42] Cook C, Heath F, Thompson R. A meta-analysis of response rates in web-or internet-based surveys. Educ Psychol Meas. 2000;60(6):821–36.

[43] Groves R. Nonresponse rates and nonresponse bias in household surveys: What do we know about the linkage between nonresponse rates and nonresponse bias? Public Opin Q. 2006;70(5):646–75.

[44] Cull WL, O’Connor KG, Sharp S, Tang SFS. Response rates and response bias for 50 surveys of pediatricians. Vol. 40, Health Services Research 2005 p. 213–26. 10.1111/j.1475-6773.2005.00350.x PMC1361134 15663710

[45] Smith W. Does Gender Influence Online Survey Participation? ERIC Doc Reprod Serv No ED 501717. 2008;1–21.

[46] Hox J. Multilevel analysis: techniques and applications. 2nd ed Mahwah, N.J.: Lawrence Erlbaum Associates; 2002. (Quantitative methodology series).

