# Supplementary material for: Perceptions of research integrity climate differ between academic ranks and disciplinary fields: Results from a survey among academic researchers in Amsterdam
Source: PLoS One. 2019 Jan 18;14(1):e0210599. doi: 10.1371/journal.pone.0210599 (PMC6338411; doi:10.1371/journal.pone.0210599)
Supplement: S1 Appendix — (PDF) [file pone.0210599.s001.pdf]

S1 Appendix. Non-response survey

NR1: We realize that you prefer not to participate in this survey.

Surveys may suffer from non-response that is non-random. To help us get a clearer picture of which type of persons prefer not to participate, we should like to learn only your rank, sex and perception of this survey as 100% safe. Would you be willing to share only that information? (Yes/no)

NR2: What is your gender? (Male/Female)

NR3: What is your academic rank? (PhD student/ Postdoc / Assistant professor / Associate professor/ Full professor / Other)

NR4: To what extent does the following statement apply to you?

I chose not to participate because the I did not feel like my data were protected (Totally disagree/Disagree/Agree nor disagree/Agree/Totally agree)
