# Supplementary material for: Perceptions of research integrity climate differ between academic ranks and disciplinary fields: Results from a survey among academic researchers in Amsterdam
Source: PLoS One. 2019 Jan 18;14(1):e0210599. doi: 10.1371/journal.pone.0210599 (PMC6338411; doi:10.1371/journal.pone.0210599)
Supplement: S1 Protocol — (PDF) [file pone.0210599.s003.pdf]

S3. Academic Research Climate Amsterdam protocol

Academic Research Climate in Amsterdam

study protocol

Table of contents

Summary

1. Project history
2. Research Team
3. Background
4. Research questions
    1. Quantitative part
    2. Qualitative part
    3. Pilot interventions

1. Methods used
    1. Ethical considerations
    2. Survey
        - 2.1. Who?
        - 2.2. How?
        - 2.3. When
        - 2.4. Preparations
    3. Focus group interviews
        - 3.1. Who?
        - 3.2. How?
        - 3.3. When
        - 3.4. Preparations
    4. Pilot interventions
        - 4.1. Who?
        - 4.2. How?
            - 6.4.2.1A Moral case deliberations
            - 6.4.2.1B Education program: mentoring the mentor
        - 4.3. When
        - 4.4. Preparations

6. **Relations of this protocol with the TWCF project**
7. **Envisioned output**
8. **Timeline**
9. **Budget**

Summary

Ensuring research integrity should be one of the key responsibilities for universities and university medical centers. Traditionally, institutions have focused on codes of conducts, procedures for handling allegations and courses on responsible conduct of research. Recently the importance of fostering a climate of research integrity receives more attention. The aim of our project is to explore and clarify the most salient aspects of the research climate in the four academic institutions in Amsterdam and to identify promising ways to promote responsible research practices. Special attention will be given to differences between academic ranks and disciplinary fields. We will assess the salient aspects of research climate by a web-based survey among all active researchers (n > 5000) using the Survey of Organizational Research Climate. The level of perceived publication pressure will be determined with the Publication Pressure Questionnaire. We will also explore the perceived frequency of occurrence of 60 research misbehavior items, what their impact is on validity of study findings and explore to what extent these misbehaviors impact the trust in science. Departing from the survey results we will determine with focus group interviews what the perceived barriers for responsible conduct of research are. Also the thoughts and perceptions of scientists on the preventability of research misbehaviors will be explored as are the solutions and interventions believed to be most effective. Subsequently we will design and try out two pilot interventions that are based on the knowledge gathered in the web-based survey and the focus group interviews: 1) regular moral case deliberation sessions in research groups, and 2) a training program for novice mentors of PhD students.

1. Project history and formation

The project Academic Research Climate in Amsterdam is a merger of two different research proposals that complement each other. The first part of the larger project called The Epistemic Responsibilities of the University, which has been granted by the Templeton World Charity Foundation (TWCF - see **Annex 1** for full description of this project). In the part of the TWCF project that is contained in this study protocol, we collect quantitative and qualitative data to investigate what aspects of the research climate promote or hinder research integrity, specifically focused on the Vrije Universiteit Amsterdam. We will develop two pilot interventions that aim to foster responsible conduct of research.
The second proposal (See **Annex 2**) that is merged in this study protocol is an extension of the TWCF project (**Annex 1)** with co-financing from the institutions collaborating in the Amsterdam Academic Alliance (AAA) and is called Research Culture in Amsterdam (**Annex 2**). This project has the same research questions as the relevant part I of the TWCF project – with the exception of the piloting of two interventions – and is in fact its extension to the three other academic institutions in Amsterdam (VU University Medical Center, Academic Medical Center and University of Amsterdam).

2. Research Team:

Core team

Lex Bouter, PhD, Professor of Methodology and Integrity, Department of Philosophy, Faculty of Humanities, Vrije Universiteit and Department of Epidemiology and Biostatistics, VU University Medical Center, Amsterdam
Joeri Tijdink, MD, PhD, postdoctoral researcher and psychiatrist, Department of Philosophy, Faculty of Humanities, Vrije Universiteit Amsterdam
Tamarinde Haven, PhD student, Department of Philosophy, Vrije Universiteit Amsterdam
Steering Committee
Rene van Woudenberg, PhD, Professor of Epistemology and Metaphysics, Department of Philosophy, Faculty of Humanities, Vrije Universiteit Amsterdam
Yvo Smulders, MD, PhD, Professor of Internal Medicine, VU University Medical Center
Gerben ter Riet, MD, PhD, Associate Professor of Epidemiology, Amsterdam Medical Center
Hanneke de Haes, PhD, Professor emeritus of Medical Psychology, Amsterdam Medical Center
Frans Oort, PhD, Professor of Methods and Statistics, University of Amsterdam
Guy Widdershoven, PhD, Professor of philosophy and medical ethics, Vrije Universiteit, Amsterdam
Brian Martinson, PhD, Senior Research Investigator, HealthPartners Institute for Education and Research, Minneapolis, Minnesota, USA

3. Background

Optimizing the validity and reproducibility of the results of scientific investigations is one of the key responsibilities of universities and university medical centers. Traditionally, universities have served these purposes by conventional efforts, such as promoting methodological rigor and rewarding successful scientists. Lately, however, it has become clear that it is also necessary to specifically foster research integrity by the promotion of responsible conduct of research (RCR) and the prevention of research misconduct (RM) and questionable research practices (QRP). To realize these aims, various interventions are available: offering education, providing facilities, issuing rules, codes, and guidelines, or influencing the research climate directly. Unfortunately, very little sound empirical evidence is available as to what the most important issues are. Also little is known about the views of scientists regarding the way the research climate promotes or hinders research integrity. This project will help to collect this evidence. We will do this within and to the benefit of our own academic institutions, but with a view to also generate generalizable insights. The main research question is:

What do scientists of the four academic institutes in Amsterdam consider to be the salient aspects of the research climate of their institution that promote or hinder research integrity, and which do they believe to be the most important barriers to responsible conduct of research (RCR) and the most promising interventions to prevent research misbehavior (RM) and questionable research practices (QRP)?

We will collect empirical evidence in the four academic institutions that collaborate in the Amsterdam Academic Alliance (AAA): University of Amsterdam, Vrije Universiteit, VU University Medical Center, and Amsterdam Medical Center.

Special attention will be given to differences between disciplinary fields (biomedical sciences, natural sciences, social sciences, and humanities) and between academic ranks (PhD students, postdoctoral fellows, assistant professors, associate professors, and full professors). Focusing the data collection on the four Amsterdam academic institutions enables intense interaction with the corresponding academic communities and an opportunity to identify promising interventions. We will analyze and report our data overall and separately for the four main disciplinary fields and academic ranks homogenous for 3 academic ranks (PhD students, postdocs plus assistant professors, and associate plus full professors). We will not do so for institutions, faculties, divisions, research institutes and departments. We will analyze similarities and differences between our data and the available findings with the questionnaires in other countries and compare the ranking results with the first study on ranking research misbehaviors (Bouter, Tijdink, Axelsen, Martinson, & ter Riet, 2016).

4. Research questions

The project will consist of three parts in which complementary parts of the main research question will be answered.

5.1 Quantitative part: web-based survey

a. What are the perceptions of academic researchers in Amsterdam regarding the organizational climate for responsible research practices both in one's general organizational setting and in one's specific department, stratified for academic rank and disciplinary field?
b. What is the level of publication pressure in the four academic institutions in Amsterdam, stratified for academic rank and disciplinary field?
c. What do scientists in the four academic institutions in Amsterdam consider to be the most detrimental research misbehaviours, stratified for academic rank and disciplinary field?

2. Qualitative part: focus group interviews

d. What are the perceived barriers for responsible conduct of research in different disciplinary fields?
e. What are the experiences and views of scientists on preventability of questionable research practices in different disciplinary fields?
f. What type of solutions and interventions can be identified that scientists expect to be the most effective, stratified for disciplinary field?

5.3 Part 3: Pilot interventions

g. How can moral case deliberations foster responsible conduct of research?
h. How can an educational program for novice mentors of PhD Students foster RCR?

6. Methods

The methods of the project are differentiated into three parts introduced above. The first part (the survey) will take place in the first year of the project. During this first year we will also start the preparations for part two and three: the focus groups at the different academic institutions that will be held in the second year of the project, and the pilot interventions in the third year of the project. Furthermore, during the first months of the project most energy and time will be used for getting formal endorsement by the four universities, the email-addresses of active scientists, and ethical approval).
The second year of the project will consist of performing, analyzing and reporting focus group interviews. In the third year we will organize the two pilot interventions. We will try to gain insight in their practical implementation and effect by collecting information at baseline and measure same variables after one year.

6.1 Ethical considerations

All participants receive an information letter by mail prior to the survey invitation. In the invitation, a link to start the actual survey is contained. The first survey item asks the respondent to actively consent by indicating that they have understood the purpose of the study and that they voluntarily take part. The study will be reviewed by two independent ethics committees from two different institutions to determine its approval. The protocol will be submitted to the Institutional Review Board (METC) of the VUmc with a view to confirm that the Medical Research Involving Human Subjects Act (WMO) does not apply and to judge whether the proposed study is compliant with the pertaining privacy legislation. Furthermore the protocol will be reviewed by the ethics committee for behavioral and movement sciences from the VU University.
Confidentiality is maintained using restricted, secure access to the data, destruction of audiotapes after transcription, and anonymous analysis of transcripts.

6.2. Web-based survey

6.2.1. Who?

TH will conduct the web-based survey under close supervision of JT. There will be a weekly meeting with TH and JT to discuss progress and for supervision. Furthermore we will have a fortnightly meeting with LB to determine progress, methods, drawbacks and results.
Furthermore there will be regular (six times per year) meetings with the full steering group to discuss methods, limitations, progress and results.

6.2.2. How?

In the first year of the project we will conduct the web-based survey to get insight in what the members of our academic community believe to be the most salient aspects of the research climate and the most important ways to improve it.
The core of the questionnaire consists of two validated instruments: the Survey of Organizational Research Climate (Martinson et al. 2013; Crain et al. 2013; Wells et al. 2014) and the Publication Pressure Questionnaire (Tijdink et al. 2013; Tijdink et al. 2014; Tijdink, Verbeke & Smulders 2014). (We have plans to improve the PPQ by re-analyzing the data and adding additional items to the questionnaire to increase construct validity.)

Additionally we will ask our respondents to rank major and minor research misbehaviors on frequency, impact, and preventability. This instrument was piloted among and discussed with 34 experts at the fourth World Conference on Research Integrity in June 2015 and surveyed among every WCRI participant of the past four World Conferences on Research Integrity (Bouter et al., 2016). Taken together the web-based survey will provide insight in how our academic community perceives a) research climate, b) publication pressure, and 3) research misbehavior. We will present the results stratified for academic rank (PhD students, postdocs & assistant professors, associate professors and full professors) and disciplinary field (biomedical sciences, natural sciences, social sciences and humanities). We will also summarize the data for each of the university faculties and the different research institutes of the university medical centers, (see table 1) , together with the corresponding overall results as a benchmark. This will enable informed discussions with a view to raise awareness and to identify actions that can be taken in the faculty or division at issue. We will only write a report per faculty/division if at least 25 respondents from that faculty/division took part in the survey to protect the participants' identity. We will invite all members of the four academic institutions in Amsterdam that are active in research (>0.2 fte) to participate in the survey. This large number of invitees (approximately 5,000) enables the combination of identity protection and detailed subgroup analyses. We also can afford to use a 100% sample because the costs of a web-based survey hardly depend on the number of participants. All invitees will get an e-mail with a link to the web-based survey. The invitational e-mail (See **Annex 3**) will also contain a link to decline participation. The latter will open a screen with a few simple questions that will enable a non-response analysis. We will formulate a strict privacy policy and provide a link to it in our invitational e-mail (**Annex 3**). Qualtrics is chosen as the online distributor of the web-based questionnaire as they have the most efficient and state of the art response techniques available. It is a professional and reliable third party that can guarantee anonymity.

For the research team it will be impossible to link records to e-mail addresses of respondents. Please note that the draft questionnaire (**Annex 4**) contains all 60 items of minor and major research misbehaviors, while each respondent will only get a random sample of 20 of these in random order. We will end the survey with demographic information on gender, academic position, faculty/division, and disciplinary field. We will aim to have a mean maximum time to complete the survey of 12 minutes.

We will use advanced marketing techniques to determine whether the invitational email is received, opened and whether the participant followed the link to the online survey.

A detailed data-analysis plan will be ready before the start of data collection (**Annex 12**).

UvA	VU	AMC	VUmc

Economics and Business	Behavioral and Movement Sciences	Cardiovascular Diseases	EMGO Intitute for Health & Care Research
Faculty of Dentistry (ACTA)	Faculty of Dentistry (ACTA)	Epidemiology and public health	IcarVU, Institute for Cardiovascular Research
Faculty of Humanities	Faculty of Humanities	Gastrointestinal Diseases and metabolic disorders	MOVE Research Institute Amsterdam
Faculty of Law	Faculty of Law	Infection and Immunity	Amsterdam Neuroscience
Faculty of Science	Faculty of Sciences	Neurological and Psychiatric Disorders	VUmc Cancer Center Amsterdam
Faculty of Social and Behavioral Sciences*	Faculty of Social Sciences	Oncology	Division VI
	Faculty of Theology	Reproduction and development	
	Economics and Business Administration		

*Table 1. Faculties (UvA and VU) and research institutes (AMC and VUmc) of the four academic institutions in Amsterdam. For more information on faculties in UvA and VU, see link & link, on research institutes in AMC and VUmc, see link & link) *Faculty of Social and behavioral Sciences consists of 3 different research institutions with a considerable number of scientists that may lead to three different reports.*

6.2.3. When?

The first part will take place in the first year of the project. See project timeline (**Annex 9**) for more details on planning.

6.2.4. Preparation

List of necessities for the start of the survey:

1. Access to all email addresses of all active scientists within the four institutions.
2. Permission of the rector's offices of the Vrije Universiteit (Prof. V. Subramaniam) and University of Amsterdam (Prof. K. Maex) and dean's offices of the VUmc (Prof. C. Polman) and AMC (Prof. J.H. Romijn) and from the ethical committees of the VUmc and by the ethics committee for behavioral sciences (the Amsterdam Institute of Social Science Research) from the university of Amsterdam (UvA) that performs an ethical review of our protocol to start the research project
3. Prepared invitational emails
4. Web-based survey (using Qualtrics) containing the Survey of Organisational Research Climate, the Publication Pressure Questionnaire, the ranking of research misbehaviors and relevant characteristics of the participants.

6.3 Qualitative part: Focus group interviews

6.3.1. Who?

The focus group interviews will be performed by different members of the research team. TH and JT will lead the group discussions when he/she has followed the necessary training in qualitative research methods. This can be organized in the first year of the project. JT has experience in conducting and analyzing focus group interviews and will supervise TH. The results will be analyzed by 3 scientists with experience in focus group study analysis to make sure the results are reliable and consistent.

2. How?

Participants

In the second year of the project we will conduct focus groups interviews (Creswell 1998; Krueger & Casey 2000; Tijdink et al. 2016) with members of our academic community to explore their views on the research culture in general and on the most important forms of RM and QRP in particular, as well the values that underlie these views. We will start from what was learned through the web-based survey, in order to deepen our understanding of what the results of the web-based survey really mean and imply for actions to be taken. The interviews will aim specifically at identifying perceived barriers to RCR and differences between academic ranks.

We will allow for differences between disciplinary fields. For that purpose we will conduct separate focus group interviews for biomedical sciences, natural sciences, social sciences, and humanities. Per disciplinary field we will first conduct 3 groups homogenous for academic rank (PhD students, postdocs plus assistant professors, and associate plus full professors), then 2 groups with mixed composition with a focus on the differences in views between the academic ranks that we identified in the homogenous focus groups. Finally we will conduct 2 focus group interviews with mixed composition of disciplinary fields but with a focus on homogeneous academic rank to determine differences among disciplinary fields. We will avoid having close colleagues in the same focus group. Together there will be (3 x 4 + 2 x 4 + 2 x 3) = 26 focus group interviews with 8 participants each. By means of intensive discussion among the participants in the focus groups, views, thoughts and opinions of researchers will be collected. Scientists are eligible to participate if they are able to speak Dutch or English, are scientifically active (they should spend at least 0.2 FTE on research) and are willing to give informed consent. Scientists are recruited with the help of the rectors' and deans' offices of the faculties (VU and UvA) and divisions (VUmc and AMC) of the participating four institutions, each of which provided the email addresses of all active scientists. We will use random selection software specially designed for this project to randomly select the participants across the different disciplines and academic ranks and sent an invitation by email explaining the purpose of the focus group interviews. This random selection is essential to prevent selection bias. If the invited participant declined participation, we randomly select a second participant of the same type from the same department, and so on, until we have 6–8 participants from different groups/disciplines/departments per focus group.

Data collection and procedure

The research team will formulate possible discussion themes about research culture beforehand that will be based on our previous survey and a pilot version of a focus group interview that we conduct with a mix of fellow scientists from the research departments of the steering committee (Philosophy, Epidemiology/Biostatistics, internal medicine). The focus group interviews will last approximately 1.5–2 hours until the point when no new or relevant information emerge (attainment of saturation). The focus groups will be chaired by an experienced group leader that will guide the discussion (JT, TH or other experienced focus group leader). Each session will be audiotaped and transcribed verbatim. After the transcription, the material will be thoroughly analyzed independently by 2-3 experienced investigators from the research team (TH, JT and TBD) with expertise in qualitative research to get in depth information of the participants' views and opinions (Meadows & Morse 2001; Mays & Pope 2000). The analyses will then be compared and discussed with the other assessors (JT, TH and 3

independent researchers) to formulate qualitative conclusions on the identified themes and to choose exemplary quotes. The results from the focus group interviews homogenous for academic ranks will inform the interview schemes for the focus group interviews with mixed composition.
A semi structured protocol (**Annex 6**), partially based on the pilot version of the focus group will be used as a structured guidance for conducting the focus group interviews. This includes information on general aspects of focus groups, an introduction to the subject, and an initial exploration of the participants' first thoughts on research integrity to increase engagement with the discussion. After this, participants will be invited to present themes they feel are relevant for the discussion on research culture and scientific integrity. From their answers, the facilitator, in consultation with the participants, prioritizes 6–9 themes. In addition, members of the research team take notes during the sessions to capture important elements.

Analysis

An inductive content analysis will be used to analyze the data. Inductive content analysis is mainly used in cases where there are no or few previous studies dealing with the subject. A deductive approach is useful if the general aim is to test a previous theory in a different situation or to compare categories at different time periods (but that is clearly not at issue for our understudied topic). By using an inductive content analysis, we read through the data looking for recurring themes. First, the entire transcripts are read and emerging themes will be coded. New themes in the transcripts will be added to the list of codes and added to the previously analyzed results. The transcripts of the focus groups will be analyzed and coded independently by three team members (JT, TH, TBD). Individual analyses of the team members were compared and discussed to achieve consensus and to increase reliability.
To check validity, participants receive a written interpretation of the focus groups in which they participated, asking them to reflect on our interpretation and to indicate if they recognized the analysis and coding. This process of coding will yield the major themes (aim for approx. 8 themes). The results of the focus groups are then compared, analyzed and interpreted by the investigators, using an inductive approach. The final result is a summary of the themes. Typical quotes are identified per theme and per scientific rank (PhD student, postdoctoral fellow/assistant professor and associate professors/full professors) to clarify the coded themes. To optimize the quality of reporting, the COREQ checklist will be used (Tong et al, 2007)

2. When?

The focus group interviews will be performed in the second year of the project. We aim to start with the project January 2017. Consequently we hope to start this part of the project January 2018.

2. Preparation

List of necessities for the start of the qualitative part**:**

1. Semi structured focus group interview protocol (**Annex 5**), partially based on the results of the qualitative part
2. Audiotape electronics
3. Focus group interview facilities/rooms in the four academic institutions
4. Invitation of randomly selected participants
5. Training of TH to lead a focus group interviews/qualitative research methods

6.4 Pilot interventions

6.4.1. Who?

We will collaborate closely with two colleagues with relevant expertise from VU Medical Center (Prof. Guy A.M. Widdershoven (part 6.4.2.1A) and Prof. Yvo M. Smulders (part 6.4.2.1B), and Prof. Widdershoven will be closely involved in the research on moral case deliberation (part 6.4.2.1A). The part of the supervision program of novice PhD mentors will be developed during the first two years of the project (part 6.4.2.1B). We will gain information from experts in the field of academic education (i.e. the education department of the VU and VUmc) for designing an effective academic teaching module.

2. How?

The findings from the focus group interviews in combination with the relevant salient results of

the quantitative part will inform two pilot interventions to be tried out *during the third year of the project* within the academic community of the Vrije Universiteit Amsterdam. Participation in both these interventions will be voluntarily. The form of these interventions is predefined, but the content is flexible and will depend on the results of the
first 2 years of the project.

6.4.2.1. Moral case deliberation

Moral awareness and an organizational climate that allows for moral learning are important preconditions for responsible conduct of research (RCR). Moral Case Deliberation (MCD) is an established intervention in health care that raises moral awareness and fosters an open culture (Van der Dam et al. 2015; Widdershoven & Metselaar, 2012). We will organize series of 5 MCDs within at least 4 research departments in different disciplinary fields on moral dilemmas related to research integrity that the researchers at issue experience themselves. The MCDs will be analyzed using thematic content analysis.

We aim to better understand the moral dilemmas that researchers encounter in their work, by uncovering the norms and values that underlie the dilemmas. Researchers are invited to participate in de MCDs via an invitational email that explains the purpose of MCDs. They are also asked to give informed consent after having had the time to ask any clarifying questions that arose from the invitational email. By participation in the MCDs, the researchers are supported when dealing with moral dilemmas and develop their moral competences. This is expected to affect the research climate within the participating departments, by stimulating the dialogue on dilemmas among researchers. Analyses of the MCDs will gain insight in the norms and values that evoke either RCR or QRP or worse.

6.4.2.2. Training program for novice PhD supervisors

Due to big fraud cases, increased awareness of research misbehavior (ranging from fraud to more subtle forms of research misbehavior) has resulted in research integrity training for PhD students as part of their educational program. However, training of the supervisors of these PhD students receives much less attention, whereas their good mentorship and role modelling are pivotal for responsible conduct of research (Bouter et al., 2016) .
In this part of the project we will focus on the crucial role of supervisors in the education of PhD students. Novice PhD mentors will be invited to participate voluntarily, but the goal of the training is to set these mentors up with necessary competences to function as a successful mentor. With a training program for novice supervisors we will develop a module that consists of online activities, face-to-face education, regular one-on-one supervision and group meetings ('intervision'). Our goal is to empower the novice supervisors for the important task of supervising PhD students with the ultimate goal to improve their skills to foster RCR in their PhD-students and in themselves.
We target specific competencies such as leadership, professionalism, collaboration, scholar skills and communication to increase research skills (van der Lee N, et al. Med Teach 2013;35:949-55.). The framework focusses on the development of the competences and skills necessary as a researcher and is primarily used to improve general competences, specific for researchers that work in a multidisciplinary environment. To evaluate this development and training we use the System of Evaluation of Teaching Qualities (SetQ) (Lombarts MJ, et al. Ned Tijdschr Geneeskd 2010;154:A1222.). This tool aims to increase faculty's insight in teaching performance of mentors and trainees and is very suitable for our project as it gives us the opportunity to empirically evaluate our educational program to gain insight in the teaching performances of our trained mentors.

2. When?

Part 3a and 3b will be performed in the third year of the project. Start +/- November 2018.

2. Preparation

List of necessities for the start of part 3:

1. Moral case deliberation discussion leaders to guide the research groups bi-monthly with detailed guideline (**Annex 7**)
2. Detailed guideline for a module on fostering RCR for novice supervisors of PhD students (**Annex 8**)
3. Facilities (rooms, logistics, etc) to support the meetings and developing the teaching program.

4. 16 Novice supervisors of PhD students that are willing to participate in our pilot intervention

5. Intervision training program (written) + teaching modules and regular meetings for young/novice supervisors
6. Research groups of the 4 disciplinary fields that are willing to participate in our second intervention (moral case deliberation)

7. Relation of this protocol with the TWCF project

Project 1 of the TWCF proposal and this research protocol are complementary in that project 1 is *conceptual* and *normative* in nature, exploring the various epistemic values that the university ought to uphold, whereas this protocol is *empirical* in nature in that it investigates for specific epistemic values—those associated with academic integrity—to what extent they are actually pursued in research groups. It stands to reason that Responsible Conduct of Research (RCR) will involve intellectual virtues of various sorts. Therefore, there is further connection with the theoretical work done in project 3 of the TWCF which scrutinizes how groups can be intellectually virtuous. Since the humanities, including theology, are also among the academic disciplines from which research groups will be selected and to which the results of this project will be presented, this project will provide some information about how research groups in the humanities operate.

8. Envisioned output

The project will not only yield the usual output in the form of articles in scientific journals, but will also inform and inspire our academic communities and the leadership ??? the four institutions that collaborate in the Amsterdam Academic Alliance (AAA). The results of the web-based survey will be summarized for each faculty and division. The findings of the web-based survey and the focus group interviews will be discussed with the colleges of deans (VU and UvA) and the councils of division chairs (AMC and VUmc). We will make short reports per faculty/research institute to inform the deans of the faculties/institutes (only if n>25). This will result in six reports for the faculties of the UvA and five reports for the faculties of the VU University.

We will also organize presentations for our academic communities about our findings, as well as panel discussions and debates about actions to be taken.
We will additionally present our findings on the national and international media and at academic conferences, like the 6th World Conference on Research Integrity.

Scientific output

- 6 peer-reviewed papers in journals such as *Science*, Nature, PNAS, *PLoS One*, *Research Integrity and Peer Review, J Empir Res Hum Res Ethics, Science and Engineering Ethics*, *American Scientist*, *Accountability in Research*
- 6 conference presentations at conferences such as the 6th *World Conference of Research Integrity*, the conference of the *International Center for Academic Integrity*, and the next version of the *REWARD Equator Conference* (http://researchwaste.net/researchwasteequator-

conference/)

- 3 op-ed articles in leading Dutch newspapers, such as *Algemeen Dagblad, De Volkskrant*, Trouw, NRC as well as international news sources and blogs, such as *The Chronicle Higher Education* (60,000) and *The New Republic* (50,000)
- Detailed guideline for the use of moral case deliberation in research groups
- Detailed protocol for a module on fostering RCR for novice supervisors of PhD students

9. Project timeline

**Qualitative part (Year 1)**		
When?	What?	Who?
1-3 months	Writing research protocol (including approval of rectors and deans offices of the 4 institutions)	JT, TH, LB
1-3 months	METC approval at VUmc and UvA	TH, JT and LB
1-3 months	Designing PhD training program	TH, JT, LB
3-6 months	Collecting and checking emailaddresses + other practical preparations from 4 institutions	TH, JT
1-6 months	Preparing and piloting Survey	TH, JT, LB, BM
7-9 months	Sending survey + 2 reminders	TH, JT
10-12 months	Data analysis + reporting to prepare focus groups for project 2	TH, JT, LB
13-24 months	Writing first manuscript	TH, JT, LB
1-12 months	Continuous trainingprogram for TH student<br>Training qualitative research methods	TH
Month 12	Evaluation of progress of TH	TH, JT, LB
**Year 2, part 2**		
1-3 months	Preparing focus group interviews	TH, JT, to be determined
1-3 months	Writing focus group protocol	TH, JT, LB
1-3 months	Preparing practicalities (invitations, location, planning) for focus group interviews	TH
1-3 months	Performing pilot focus group and writing semistructured protocol	TH, JT
4-9 months	Conducting focus group interviews	TH, JT
7-9 months	Transcribing interviews	TH
10-18 months	Writing manuscript	TH, JT, LB
10-12 months	Preparing intervention project + writing moral case deliberation protocol	TH, JT, LB, GW
10-12 months	Writing teaching module<br>/protocol for novice supervisors	TH, JT, LB, (evt. External help)
**Year 3: Part 3a and b, intervention studies**		

1-3 months	Completing moral case deliberation (MCD) protocol (**Annex 7**)	TH, JT, LB, GW
1-3 months	Completing novice supervisors protocol (**Annex 8**)	TH, JT, LB, (external)
1-3 months	Organizing/planning intervention program with MCD and intervention groups	TH, JT, LB
1-3 months	Organizing supervisors training protocol	TH, JT, LB
4-9 months	Conducting MCD and supervisors protocol	TH, JT, LB
10-12 months	Analyzing results of intervention study (see **Annex** 7 for complete description of analysis and expected results)	TH, JT, LB
10-12 months	Writing 2 manuscripts of intervention studies	TH, JT, LB
10-12 months	Completing reports, conducting process evaluation report with recommendations	TH, JT, LB
	**Year 4: Writing PhD thesis**	
1-12 months	Extension time for publications (see above)	TH, JT, LB
1-12 months	Extended time to finalize quantitative, qualitative and intervention part.	TH, JT, LB
1-12 months	Writing dissertation	TH, JT and LB
1-12 months	Writing 2 papers with the other team members of other projects of the TWCF (**Annex 1**) to merge and combine the results of this project with the other projects.	JT, LB, RvW, RP, JdR

10. Budget

— PhD student (1.0 fte for 4 years)
The 4th year is for finishing the PhD thesis and the manuscripts of the articles based on the AAA project. Furthermore, in this last year a systematic review or a psychometric study on one of the three instruments used will be performed.
— Postdoc (0.4 fte for 4 years)
— Other costs (75 K€)2
PhD training program (12K€), Conference meetings + travel costs postdocs and PhD (3 years x 6k€ = 18k€), chairs and data-analysts focus group interviews (20 K€), external location and catering for focus group interviews (10 K€), and travel budget for postdoc and US partner (Brian Martinson) (15 K€)

References

1. Bouter LM. Perverse incentives or rotten apples? Accountability in Research 2015; 22: 148–61
2. Martinson BC, Thrush CR, & Crain AL. 2013. Development and validation of the Survey of Organizational Research Climate. Sci Eng Ethics 2013; 19: 813–34
3. Crain AL, Martinson BC, Thrush CR. Relationships between the Survey of Organizational Research Climate and self-reported research practices. Sci Eng Ethics 2013; 19: 835-40

4. Wells JA, Thrush CR, Martinson, BC, May, TA, Stickler, M, Callahan, EC, Klomparens KL 2014. Survey of organizational research climate. J Emp Res Hum Res Ethics 2*015;* 9: 72–88
5. Tijdink JK, Smulders YM, Vergouwen AC, de Vet HC, Knol DL 2014. The assessment of publication pressure in medical science: validity and reliability of a Publication Pressure Questionnaire (PPQ). Qual Life Res 2014; 23: 2055–62
6. Tijdink, JK, Vergouwen AC, Smulders YM. 2013. Publication pressure and burn out among Dutch medical professors: a nationwide survey. PLoS One 2013; 8: e73381
7. Tijdink JK, Verbeke R, Smulders YM 2014. Publication pressure and scientific misconduct in medical scientists. J Emp Res Hum Res Ethics 2014; 9: 64–71
8. Creswell JW. Qualitative inquiry and research design: choosing among five traditions. Thousand Oaks: Sage; 1998
9. Krueger RA, Casey MA. Focus groups: a practical guide for applied research. 3rd ed. Thousand Oaks CA: Sage Publications; 2000
10. Tijdink JK, Schipper K, Bouter LM, Maclaine Pont P, de Jonge J, Smulders YM. Biomedical scientists' view on publication culture: a focus group study. *BMJ Open* 2016;6:e008681
11. Mays N, Pope C. Qualitative research in health care. Assessing quality in qualitative research. BMJ 2000; 320: 50-2
12. Meadows LM, Morse JM. Constructing evidence within the qualitative project. In: Morse JM, Swanson JM, Kuzel AJ, editors. The Nature of qualitative evidence. Thousand Oaks, CA: Sage publications; 2001. p. 187-200
13. Tong D, Sainsbury P, Craig J. Consolidated criteria for reporting qualitative research (COREQ): a 32-item checklist for interviews and focus groups. International Journal for Quality in Health Care. 2007. Volume 19, Number 6: pp. 349 – 357)
14. Bouter, L. M., Tijdink, J., Axelsen, N., Martinson, B. C., & ter Riet, G. (2016). Ranking major and minor research misbehaviors: results from a survey among participants of four World Conferences on Research Integrity. *Research Integrity and Peer Review, 1*(1). doi:10.1186/s41073-016-0024-5
