# Supplementary material for: Perceptions of research integrity climate differ between academic ranks and disciplinary fields: Results from a survey among academic researchers in Amsterdam
Source: PLoS One. 2019 Jan 18;14(1):e0210599. doi: 10.1371/journal.pone.0210599 (PMC6338411; doi:10.1371/journal.pone.0210599)
Supplement: S1 Table — (PDF) [file pone.0210599.s004.pdf]

**Modification for current study**
How effectively do the available educational opportunities at your university teach staff/researchers about responsible research practices (e.g., lectures, seminars, web-based courses)?<br>How committed are the senior administrators at your university/medical centre/institution (e.g., deans, members of the executive board) to supporting responsible research?<br>How effectively do the senior administrators at your institution (e.g., deans, executive board) communicate high expectations for research integrity?<br>If you needed to report a case of suspected research misconduct, how confident are you that you would know where to turn?<br>How difficult is it to conduct research in a responsible manner because of insufficient access to human resources such as statistical expertise, technical support or administrative support?<br>How committed are supervisors in your department to talking with supervisees about key principles of research integrity?<br>How consistently do supervisors communicate to their supervisees clear performance (e.g. what is expected to earn authorship, proper citation and correct referencing)?<br>How consistently do research practices in your department follow established institutional policies regarding research?<br>How respectfully do supervisors treat supervisees?<br>How committed are people in your department to maintaining data integrity (appropriate handling of data from its collection to how its archived for potential future use) and data confidentiality?<br>How available are supervisors to their supervisees?
