# Supplementary material for: Perceptions of research integrity climate differ between academic ranks and disciplinary fields: Results from a survey among academic researchers in Amsterdam
Source: PLoS One. 2019 Jan 18;14(1):e0210599. doi: 10.1371/journal.pone.0210599 (PMC6338411; doi:10.1371/journal.pone.0210599)
Supplement: S2 Table — Clustering refers to situations where there is non-independence of observations in the data, resulting in “design effects” or “intraclass-correlations” (ICCs) in the data. In our study, respondents are clustered (or nested) in departments, that are again nested within disciplines that are themselves nested within institutions introducing dependence in the data on different levels. Inference on regression coefficients needs to take this dependence into account. Ignoring the clustering in the analyses yields estimates for the standard errors for the betas that are too small, and hence, will also result in p-values that are too small (and increase of type I-errors). For reasons of privacy, data concerning affiliation of the respondents was not available and for this reason a standard multilevel analysis correcting for clustering could not be performed. We therefore used a linear regression with a post-hoc correction of the SE’s of the beta’s using an estimate for the Variance Inflation Factor. By correcting the SE’s, we get some indication of whether the associations we found are still there had we taken clustering into account [46]. The authors of the Wells et al. [22] study calculated their ICC institute (see ICC institute in the formulae below) for us. This way we could compute the Variance Inflation Factor as a correction for the sample size we used to estimate our SE’s. We re-calculated the t-statistics, using adjusted degrees of freedom and adjusted SE’s, to assess whether the associations we found between rank or discipline and the SOuRCe subscales are also detected when clustering is taken into account. The calculations are according to the following formulas: VIF=1+(m-1)*ICCinstitute CorrectedSE=SD/(n/VIF) t=βCorrectedSE Df=NVIF-p-1 The left column describes the relevant SOuRCe subscale with the effective N, the second column the VIF value based on the ICC for Institute from the Wells et al. study, then the adjusted t-scores and significance level of the corre [file pone.0210599.s005.pdf]

**SOuRCe Subscale (*N*)**	**VIF**	**Adjusted T score (*df*)**	***p* value**	**Effect**
Integrity Norms (*1009)*	1.884			Association no
$\beta\ PhD\ vs.associate\ \&\ full\ prof.$		1.620 (531*)*	.106	longer significant
Integrity Socialization (*1018*)	1.354			
$\beta\ PhD\ vs.associate\ \&\ full\ prof.$		-5.209 (*748*)	<.001	No effect on
$\beta\ postdoc\ \&\ asis.vs.asso.\ \&\ full\ prof.$		-4.454 (*748)*	<.001	association
Integrity Inhibitors (*1020*)	6.049			
$\beta\ PhD\ vs.associate\ \&\ full\ prof.$		-1.259 (*165*)	.210	Association no
$\beta\ postdoc\ \&\ asis.vs.asso.\ \&\ full\ prof.$		-.0897 (*165*)	.371	longer significant
RCR Resources (*985*)	2.175			
$\beta\ PhD\ vs.associate\ \&\ full\ prof.$		-2.576 (*446*)	.011	No effect on
$\beta\ postdoc\ \&\ asis.vs.asso.\ \&\ full\ prof.$		-2.934 (*446*)	.003	association
Expectations (*1011*)	1.176			
$\beta\ PhD\ vs.associate\ \&\ full\ prof.$		-2.661 (*856*)	.007	No effect on
$\beta\ postdoc\ \&\ asis.vs.asso.\ \&\ full\ prof.$		-4.120 (*856*)	<.001	association
Supervisor-Supervisee relations (*999*)	1.783			
$\beta\ PhD\ vs.associate\ \&\ full\ prof.$		-3.436 (*557*)	.006	No effect on
$\beta\ postdoc\ \&\ asis.vs.asso.\ \&\ full\ prof.$		-.3062 (*557*)	.002	association
