# Supplementary material for: Perceptions of research integrity climate differ between academic ranks and disciplinary fields: Results from a survey among academic researchers in Amsterdam
Source: PLoS One. 2019 Jan 18;14(1):e0210599. doi: 10.1371/journal.pone.0210599 (PMC6338411; doi:10.1371/journal.pone.0210599)
Supplement: S3 Table — Clustering refers to situations where there is non-independence of observations in the data, resulting in “design effects” or “intraclass-correlations” (ICCs) in the data. In our study, respondents are clustered (or nested) in departments, that are again nested within disciplines that are themselves nested within institutions introducing dependence in the data on different levels. Inference on regression coefficients needs to take this dependence into account. Ignoring the clustering in the analyses yields estimates for the standard errors for the betas that are too small, and hence, will also result in p-values that are too small (and increase of type I-errors). For reasons of privacy, data concerning affiliation of the respondents was not available and for this reason a standard multilevel analysis correcting for clustering could not be performed. We therefore used a linear regression with a post-hoc correction of the SE’s of the beta’s using an estimate for the Variance Inflation Factor. By correcting the SE’s, we get some indication of whether the associations we found are still there had we taken clustering into account [46]. The authors of the Wells et al. [22] study calculated their ICC institute (see ICC institute in the formulae below) for us. This way we could compute the Variance Inflation Factor as a correction for the sample size we used to estimate our SE’s. We re-calculated the t-statistics, using adjusted degrees of freedom and adjusted SE’s, to assess whether the associations we found between rank or discipline and the SOuRCe subscales are also detected when clustering is taken into account. The calculations are according to the following formulas: The left column describes the relevant SOuRCe subscale with the effective N, the second column the VIF value based on the ICC for Institute from the Wells et al. [22] study, then the adjusted t-scores and significance level of the corrected association is given. The final right column concludes what the i [file pone.0210599.s006.pdf]

SOuRCe Subscale (*N*)	VIF	Adjusted T score (*df*)	*p* value	Effect
Regulatory Quality (*670*)	1.835			
β *humanities vs. biomedicine*		-2.558 (*359*)	.011	No effect on association
β *humanities vs. natural sciences*		-1.760 (*359*)	.079	
β *humanities vs. social sciences*		-2.314	.021	
Expectations (*1011*)	1.176			
β *humanities vs. biomedicine*		-3.728 (*853*)	<.001	No effect on association
β *humanities vs. natural sciences*		-3.907 (*853*)	<.001	
